# Thermal Inactivation of African Swine Fever Virus in Swill

**DOI:** 10.3389/fvets.2022.906064

**Published:** 2022-06-06

**Authors:** Suphachai Nuanualsuwan, Tapanut Songkasupa, Prakit Boonpornprasert, Nutthakarn Suwankitwat, Walaiporn Lohlamoh, Chackrit Nuengjamnong

**Affiliations:** ^1^Department of Veterinary Public Health, Faculty of Veterinary Science, Chulalongkorn University, Bangkok, Thailand; ^2^Department of Veterinary Public Health, Faculty of Veterinary Science, Center of Excellence for Food and Water Risk Analysis (FAWRA), Chulalongkorn University, Bangkok, Thailand; ^3^Virology Laboratory, National Institute of Animal Health, Bangkok, Thailand; ^4^Department of Animal Husbandry, Faculty of Veterinary Science, Chulalongkorn University, Bangkok, Thailand

**Keywords:** heat treatment, inactivation, African swine fever virus, swill, *D*_T_

## Abstract

The indirect transmission of the African swine fever virus (ASFV) is through contaminated fomite, feed ingredients, pork- and pig-derived products, including swill, as ASFV is highly stable within suitable organic material. Some previous studies have indicated that ASFV outbreaks were associated with swill feeding, particularly in smallholder pig farms. These outbreaks emphasize the significance of the appropriate heat treatment of swill to eliminate ASFV residual titer. The World Organization for Animal Health (OIE) recommended the heat treatment of swill at a temperature of at least 90°C for at least 60 min, with continuous stirring, while the Food and Agriculture Organization (FAO) recommended heat treatment at 70°C for 30 min. The lack of scientific evidence regarding ASFV inactivation by heat treatment of swill leads to such inconsistent recommendations. Therefore, the objectives of this study were to assess the thermal inactivation of ASFV in three swill formulae and to develop a *D*_T_ model to predict *D*_T_ at some other inactivation temperatures. The significant reduction of ASFV in swill occurred at temperatures as low as 60°C. *D*_T_ or decimal reduction time (DRT) is defined as the time required to reduce the virus titer by 1 log, and this was also used as a comparative index of heat resistance. The mean *D*_60_, *D*_70_, *D*_75_, and *D*_80_ of ASFV in three swill formulae were in the ranges 23.21–33.47, 5.83–10.91, 2.15–2.22, and 1.36–1.47 min, respectively. These *D*_T_ could be widely used for any nutritive composition of swill other than the three swill formulae in this study since there was no statistical difference of all *D*_T_ of ASFV across three swill formulae. Based on *D*_70_ and the predicted *D*_90_ from the *D*_T_ model in this study, including the highest ASFV titer in pork products, the calculated inactivation times at 70 and 90°C were 119 and 4 min, respectively.

## Introduction

African swine fever virus (ASFV) belongs to the family *Asfarviridae* and is an enveloped double-stranded DNA virus with a genome between 170 and 194 kilobase pair in a virion diameter of 172–191 nm (1). ASFV causes a contagious disease in wild boar and domestic pigs. The high morbidity and mortality rates of African swine fever (ASF) cause serious economic and production losses worldwide. The direct transmission route is through contact with sick pigs. The indirect transmission route is through contaminated fomites, feed ingredients, consumption of pork or pig-derived products, including when present in swill since ASFV is extremely stable in various conditions, and, particularly, pork products (2, 3).

ASFV inactivation is temperature dependent. ASFV stability in manure was varied by the storage temperature as a recent study has demonstrated that ASFV persists for up to 8 days at 4°C and 4 days at 37°C (4), while the effective heat treatment of ASFV was recommended at 56°C for 70 min or 60°C for 20 min (2). However, low or freezing temperatures preserve ASFV for a long period of time (5). ASFV is persistent in frozen pork and blood for more than 2 years and 6 years, respectively (6, 7). Since ASFV is highly stable in pork-derived products, ASFV could be transmitted whenever such contaminated products were used as swill (3).

Some previous studies have indicated that ASFV outbreaks were caused by swill feeding in pig farms (8–10). In 2012, an epidemiological study along the Kenya–Uganda border indicated that the major risk factor highly associated with ASF occurrence was feeding of pigs with swill in smallholder pig farms (*p* <0.024). In 2018, a Chinese laboratory reported the isolation of ASFV from pig farms where pigs were fed with a table scrap. The ASFV from this outbreak belonged to the genotype II group and was perfectly matched with Georgia (2007/1), Krasnodar (2012), Irkutsk (2017), and Estonia (2014) isolated in Georgia, Russia, and Estonia based on the p72 gene (9). In 2019, the association of outbreak and the swill feeding practice was reported in Mongolia. This outbreak involved 83 pig smallholders and resulted in almost 3,000 dead or culled pigs across 7 provinces. In Mongolia, imported pork products were common among food premises because of the short domestic supply. These imported pork products act as a source of ASFV in conjunction with ASFV resistant to heat treatment, and then the residual ASFV could act as a primary source of ASFV introduction to Mongolia. A common practice among pig smallholders is to feed their pigs with table scraps or food waste without sufficient heat treatment, although ASFV infection in swill feed has been detected. Swill feeding was not prohibited in Mongolia due to socioeconomic reasons. This outbreak emphasizes the significance of the appropriate heat treatment of swill feed to eliminate ASFV residual titer (8).

According to Article 15.1.22. of the Terrestrial Animal Health Code, inactivation of ASFV in swill is achieved by keeping a temperature of at least 90°C for at least 60 min with continuous stirring (11), while the FAO recommended heat treatment of swill to inactivate ASFV at a temperature of 70°C for 30 min (3). The lack of scientific evidence regarding ASFV inactivation by heat treatment of swill leads to such inconsistent recommendations of heat treatment to completely inactivate swill contaminated by ASFV. Therefore, the objectives of this study were to assess the thermal inactivation of ASFV in different protein or fat contents of swill feed within the range of temperatures recommended by international organizations and to develop a *D*_T_ model to predict *D*_T_ of some other inactivation temperatures.

## Materials and Methods

### Cell Preparation

Primary swine macrophages were aseptically collected from 24-week-old crossbred pigs in which the absence of PCV2, CSFV, PRRSV, and ASFV was confirmed by polymerase chain reaction (PCR) assay. Peripheral blood morphonuclear cells (PBMCs) were prepared from defibrinated swine blood as previously described (2). The cells were cultured in autogenous pig serum for maturation, and then, after 3–4 days, monocyte-derived macrophage (MDM), that is macrophage-like round cells, was proliferated on a vessel surface. The cells were continually cultured in an RPMI−1640 (Gibco, Waltham, MA, USA) culture medium containing 10% fetal bovine serum (Sigma-Aldrich, St. Louis, MO, USA) and supplemented with antibiotic–antimycotic solution (Gibco, Waltham, MA, USA).

### ASFV Titration

The ASFV isolates (Asian epidemic strain, genotype II) were originated from pork products confiscated from international tourists during 2018 and 2020. The ASFV stocks (ASFV-NIAH-BL01–05) for the inactivation studies were routinely maintained and titrated in PBMCs culture and stored in aliquots at −80°C until use. All experiments with ASFV were performed at biosafety Level 3 at the National Institute of Animal Health.

The viral titers of supernatants from each feed ingredient matrix spiked with ASFV isolates were determined by PBMC cell cultures. Approximately, 1.5 × 10^6^ cells/well in 96-well plates were seeded in each well for 3–4 days before the assay. Fifty microliters of a 10-fold serial solution of samples were inoculated into the wells in quadruplicate, and they were incubated in a CO_2_ incubator at 37°C for 5–7 days. The presence of haemadsorption (HAD) was examined under the microscope, and the 50% HAD infectious dose per ml (HAD_50_/ml) was calculated using the Reed and Muench method (12).

### Swill Feed

Three swill formulae in this study were autoclaved at 121°C and 15 lb/inch^2^ pressure for 15 min to eliminate ASFV contamination. The proximate analysis of swill formulae was performed 3 times to determine the average and standard deviations of crude fiber, crude fat, moisture, total carbohydrate (excluding fiber), ash, and crude protein. The results are shown in Table 1.

**Table 1:** Proximate analysis of three swill formulae.

**Swill composition**	**Percent (w/w)^a^**		
	**Swill formula 1**	**Swill formula 2**	**Swill formula 3**
Crude fiber	5.42 ± 1.23	0.59 ± 0.21	0.50 ± 0.11
Crude Fat	0.98 ± 0.14	2.99 ± 0.42	4.43 ± 0.69
Moisture	0.98 ± 0.14	72.82 ± 2.42	70.44 ± 0.21
Total Carbohydrate	15.21 ± 2.49	25.38 ± 2.21	21.71 ± 0.12
Ash	0.57 ± 0.04	1.82 ± 0.42	0.76 ± 0.05
Crude Protein	3.22 ± 0.37	7.96 ± 0.25	2.74 ± 0.13

a * Average ± SD*.

### Thermal Inactivation

Five grams of ASFV-free swill were added to a 50-ml centrifuge tube. Before the addition of ASFV, the feed ingredients were preheated at 60, 70, 75, and 80°C in digitally controlled Heating Cooling Drybath (Thermo Fisher Scientific, Waltham, MA, USA). The initial infectious ASFV suspension had a titer of Thermos 5.0 log HAD_50_/ml. In this study, we prepared six samples per set per temperature. Each set consisted of triplicate samples spiked with 500 ml of ASFV; a positive control without any treatment (ASFV suspension), a positive non-treated control (a swill sample spiked with ASFV), and a negative non-treated sample control (a swill sample without ASFV, control at time zero). The inactivation temperature was monitored with a thermocouple. After the respective treatments, the samples were immediately immersed in an ice bath for 30 min to stop the reaction. The samples were added and mixed with 0.5 ml of a cell culture medium (RPMI−1640). The mixture was centrifuged, harvested, and stored at −80°C until the residual virus was titrated.

### Inactivation Curve

The rate of ASFV inactivation is assumed to follow first-order kinetics (13, 14). At a constant inactivation temperature, a linear curve of inactivation rate is fitted to the logarithmic scale of residual ASFV titers as a function of the inactivation time. *D*_T_ or decimal reduction time (DRT) is defined as the time required to reduce the virus titer by 90% (1–log reduction) at a constant inactivation temperature. *D*_T_ was also used as a comparative index of heat resistance across several inactivation temperatures for an equivalent 1–log reduction. *D*_T_ is determined by the negative reciprocal of the slope of the inactivation curve at an inactivation temperature *T* as shown in the following equation:


(1) $$\begin{array}{r} \log N_{t}=-\frac{t}{D_{T}}+\log N_{0} \end{array}$$

where *N*_*t*_and *N*_0_ are the ASFV titers at inactivation times *t* and zero, respectively.

### *D*_T_ Model

The DRT curve is fitted to a series of log *D*_T_ values corresponding to their inactivation temperatures. The linear equation of such a DRT curve is fitted to the multiple logarithmic *D*_T_ values as a function of inactivation temperature (15). This linear equation acts as the predicted *D*_T_ model where the independent and dependent variables are inactivation temperature and *D*_T_, respectively. Analogous to *D*_T_, *z* is the negative reciprocal of the slope of the DRT curve. Therefore, the *z* value is the temperature required to change *D*_T_ by 1–log. The *D*_T_ of an inactivation temperature could be predicted by the *D*_T_ model using the *z* value and the y-intercept of the fitted linear equation (the DRT curve) as shown in the following equation:


(2) $$\begin{array}{r} \log D_{T}=\;-\frac{T}{z}+y-intercept \end{array}$$

where

*D*_T_ is the *D* of ASFV at an inactivation temperature *T*

*z* is the negative reciprocal of the slope.

### Statistical Analyses of the Inactivation Curve and DRT Curve

To determine the statistical significance of the inactivation curve of ASFV in swill at each inactivation temperature, regression analysis using an *F*-test with a level of significance of 0.05 was applied to the inactivation curve, i.e., the slope of the inactivation curve as the regression coefficient was significantly different from zero. Furthermore, to determine the statistical significance of the DRT curve of ASFV in each swill formula, the slope of the DRT curve as the regression coefficient was tested for the difference from zero to see whether *D*_T_ was temperature dependent or not. The goodness-of-fit (*gof* ) values of both the inactivation curve and the DRT curve were determined from the correlation coefficient (*r*^2^) and the root mean square error (RMSE) (16). The temperature effect and swill formula effect for ASFV were simultaneously determined by two-way analysis of variance (ANOVA). Once ANOVA indicated a significant difference, Tukey's multiple comparisons were performed to determine the pair-wise differences of either inactivation temperatures or swill formulae. IBM^®^ SPSS^®^ Statistics version 22 software (SPSS Inc., Chicago, IL, USA) was used to perform statistical analyses.

### Correlation of Swill Composition and *D*_T_

Pearson's correlation coefficient (*r*) was used to evaluate the correlation of some nutritive compositions of swill and the heat resistance of ASFV in swill in terms of *D*_T_.

## Results

### ASF Virucidal Activity by Heat Treatment

**Figure 1:**
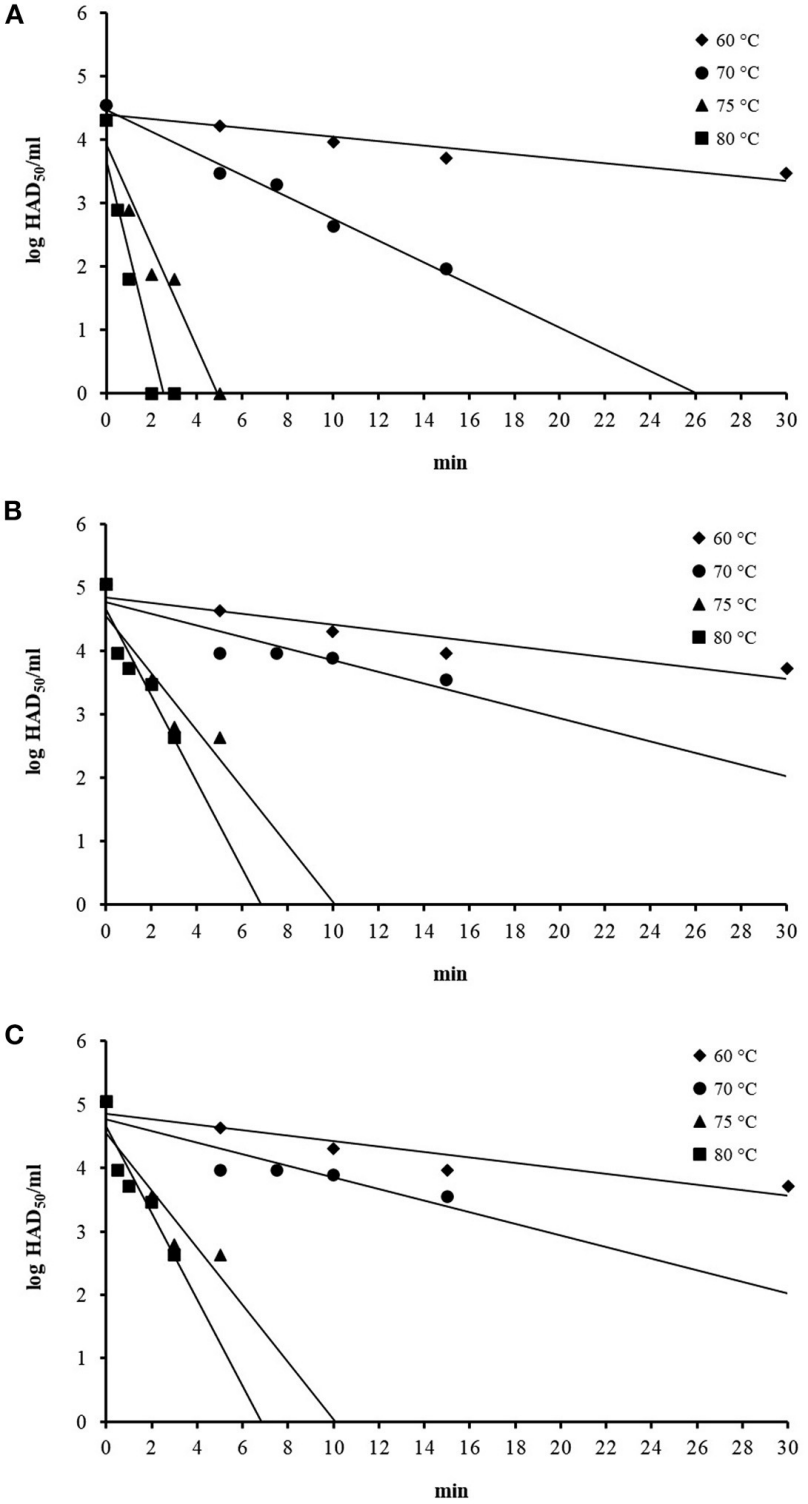
Thermal inactivation of ASFV at 60, 70, 75, and 80°C in swill formula 1 **(A)**, swill formula 2 **(B)**, and swill formula 3 **(C)**.

Immediately after mixing the ASFV working suspension with the swill in the centrifuge tubes, the initial ASFV titers in three swill formulae were 4–5 log HAD_50_/ml at time zero. The ASFV titers, after being subjected to inactivation temperatures at 60, 70, 75, and 80°C, gradually dropped with different rates, depending upon the inactivation temperatures (Figure 1). In general, the ASFV titer dropped faster at a higher inactivation temperature for all three swill formulae. The mean ASFV titer at 60°C was slowly reduced by only 1 log after 30-min inactivation time, while the mean ASFV titer at 80°C was sharply reduced more than 2. log after only 3-min inactivation time. The thermal inactivation of ASFV in swill appears to be temperature dependent (Figure 1).

### *D*_T_ of ASFV From Thermal Inactivation

The linear equation, as the inactivation curve, was fitted to mean log titer of ASFV (*N*_*t*_) on the y-axis as a function of inactivation time on the x-axis (*t*) as shown in Figure 1. Upon obtaining the inactivation curve, the slope of this inactivation curve gives the inactivation rate of ASFV by heat treatment in the swill formulae. The mean *D*_T_, inactivation curves and *gof* of ASFV in three swill formulae across four inactivation temperatures are shown in Table 2. The mean *D*_60_, *D*_70_, *D*_75_, and *D*_80_ of all swill formulae are in the ranges 23.21–33.47, 5.83–10.91, 2.15–2.22, and 1.36–1.47 min, respectively. For three swill formulae, the ASFV inactivation curves across four inactivation temperatures are statistically significant (*p* < 0.05). This indicates the virucidal activity of heat treatment against ASFV as low as 60°C.

**Table 2:** *D*_T_ and inactivation curves of ASFV in swill formulae at 60, 70, 75, and 80°C.

**Swill formula**	**Temp (**°**C)**	***D*** **_T_ (min)^a^**	**Inactivation curve^**b**^**	***gof*** ^**c**^		***p*** **-value**
				***r*** **^2^**	**RMSE**	
1	60	28.96 ± 4.25	log *N_*t*_* = −0.03*t* + 4.39	0.84	0.17	<0.001
	70	5.83 ± 0.52	log *N_*t*_* = −0.17*t* + 4.47	0.93	0.25	<0.001
	75	2.17 ± 0.05	log *N_*t*_* = −0.46*t* + 3.55	0.66	0.61	<0.001
	80	1.41 ± 0.04	log *N_*t*_* = −0.71*t* + 3.44	0.60	0.67	<0.001
2	60	23.21 ±2.96	log *N_*t*_* = −0.04*t* + 4.85	0.82	0.22	<0.001
	70	10.91 ±5.25	log *N_*t*_* = −0.09*t* + 4.77	0.72	0.30	<0.001
	75	2.22 ± 0.12	log *N_*t*_* = −0.45*t* + 4.54	0.81	0.40	<0.001
	80	1.47 ± 0.10	log *N_*t*_* = −0.68*t* + 4.65	0.86	0.31	<0.001
3	60	33.47 ±5.81	log*N_*t*_* = −0.03*t* +4.41	0.81	0.16	<0.001
	70	10.71 ± 0.91	log*N_*t*_* = −0.09*t* + 4.45	0.89	0.17	<0.001
	75	2.15 ± 0.04	log*N_*t*_* = −0.47*t* +3.71	0.77	0.47	<0.001
	80	1.36 ± 0.14	log *N_*t*_* = −0.73*t* + 3.84	0.85	0.35	<0.001

a *Mean ± S.D*.

b *ASFV titer (log N_t_) at inactivation time t (min)*.

c *Goodness-of-fit*.

The results of Tukey's multiple comparisons of *D*_T_ in swill formulae at 4 inactivation temperatures are shown in Table 3. Overall, the inactivation temperatures are negatively correlated with *D*_T_; as the inactivation temperature increases, the mean *D* decreases. As expected, the mean *D*_60_ of ASFV in all swill formulae is highest, and this is followed by *D*_70_, *D*_75_, and *D*_80_, respectively (*p* < 0.05), i.e., a higher inactivation temperature possesses a lower *D*_T_ and *vice versa*. The significant differences of *D*_T_ across inactivation temperatures indicate the temperature effect against ASFV in swill. Heat treatment of the swill at a higher temperature could reduce the ASFV titer faster than that of the swill at a lower temperature. Therefore, one can shorten the heat treatment time by increasing the heat treatment temperature to achieve the same amount of ASFV log reduction in swill. There was no significant difference of all *D*_T_ among the three swill formulae. This indicates that different combinations of protein, fat, or carbohydrate in the swill did not affect the virucidal activity against ASFV at any heat treatment temperatures in this study.

**Table 3:** Mean *D*_60_, *D*_70_, *D*_75_, and *D*_80_ (min) of ASFV across three swill formulae.

**Swill formula**	***Mean D*** **_T_ (min)**			
	***D*** **_60_**	***D*** **_70_**	***D*** **_80_**	***D*** **_85_**
1	28.96^A,a^	5.83^A,b^	2.17^A,b,c^	1.41^A,d^
2	23.21^A,a^	10.91^A,b^	2.22^A,c^	1.47^A,d^
3	33.47^A,a^	10.71^A,b^	2.15^A,c^	1.36^A,d^

*In the column-wise comparison, the mean D_T_ with different letters implies that there are statistically significant differences (p < 0.05) among the different feed ingredients for the same inactivation temperature (letters A and B). In the row-wise comparison, mean D_T_ with different letters implies that there are statistically significant differences (p < 0.05) among the different inactivation temperatures for the same feed ingredient (letters a through d)*.

### *D*_T_ Model

**Figure 2:**
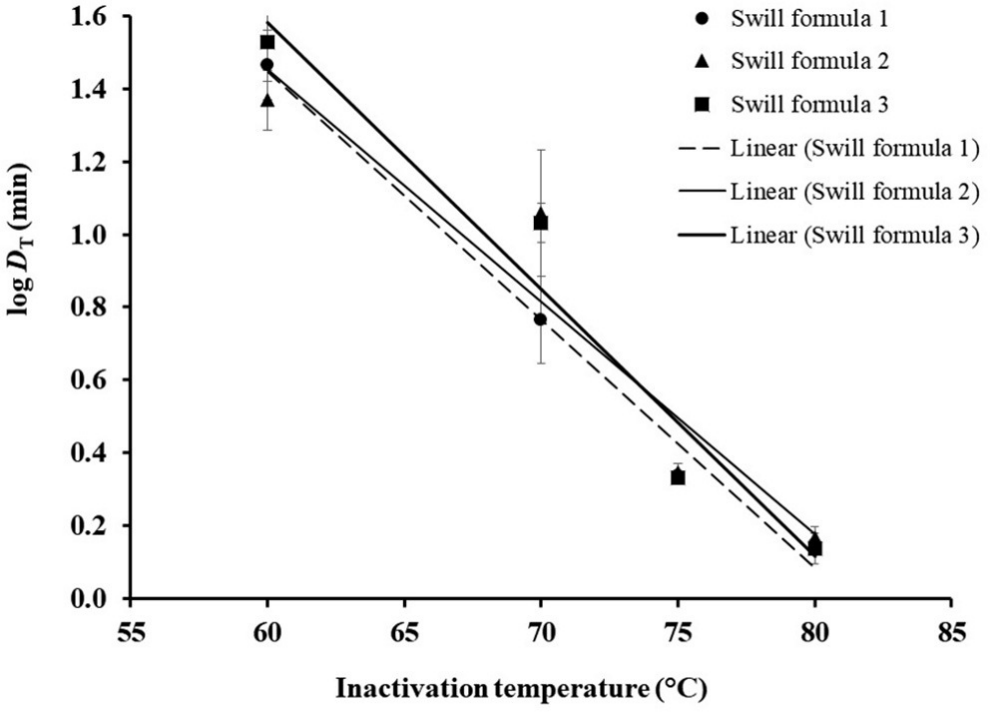
DRT curves were fitted to log *D*_T_ of ASFV in three swill formulae. The error bar indicates the standard deviation of log *D*_T_.

Based on *D*_T_ in Table 2, the DRT curves were drawn from the log *D*_T_ of ASFV in swill formulae on the *y*-axis against the inactivation temperatures on the *x*-axis (Figure 2). The *z* value is determined by the negative reciprocal of the slope of the DRT curve of each swill formula. The mean and 95% confidence interval of *z* values and the *D*_T_ models of 3 swill formulae are shown in Table 4. The slope of the DRT curve as the regression coefficient is significantly different from zero (*p* < 0.001), indicating that the *D*_T_ is temperature dependent. The *gof* of all *D*_T_ models indicates that such a *D*_T_ model was well-fitted to the DRT curve for all inactivation temperatures tested. Therefore, these *D*_T_ models well predict *D*_T_ of some other inactivation temperatures.

**Table 4:** Comparison of *z* values and *D*_T_ models of ASFV in 3 swill formulae.

**Swill**	***z*** **value (°C)**		***D*** **_T_ model^a^**	***gof***		***p*** **-value**
	**Mean**	**95% CI**		***r*** **^2^**	**RMSE**	
Formula 1	14.71	13.50–16.15	log *D*_T_ = – $\left(\frac{T}{14.71}\right)+$ 5.52	0.98	0.07	<0.001
Formula 2	15.68	12.54–20.92	log *D*_T_ = – $\left(\frac{T}{15.68}\right)$ + 5.28	0.89	0.18	<0.001
Formula 3	13.63	11.71–16.32	log *D*_T_ = – $\left(\frac{T}{13.63}\right)$ + 5.98	0.95	0.14	<0.001

a *log D_T_ (min) for the unknown inactivation temperature T (°C)*.

### Correlations of Swill Compositions and *D*_T_

Pearson correlation coefficients (*r*) of ash, crude fiber, crude fat, moisture, crude protein, and total carbohydrate with *D*_60_, *D*_70_, *D*_75_, and *D*_80_ of ASFV are shown in Table 5.

**Table 5:** Pearson correlation coefficients (*r*) of swill compositions and *D*_T_ of ASFV in swill.

**Swill composition**	**Correlation coefficient (** ***r*** **)**			
	***D*** **_60_**	***D*** **_70_**	***D*** **_75_**	***D*** **_80_**
Crude fiber	0.04	−0.71	−0.10	−1.06
Crude fat	0.27	0.59	−0.08	−0.12
Moisture	−0.07	0.72	0.12	0.08
Total carbohydrate	−0.32	0.72	0.25	0.22
Ash	−0.63	0.56	0.40	0.39
Crude protein	−0.71	0.44	0.43	0.43

The *r* of swill compositions and *D*_60_ of ASFV are inconsistent with those of *D*_70_, *D*_75_, and *D*_80_ in that the sign of *r* of *D*_60_ is the opposite to the sign of *r* of other *D*_T_ except *D*_70_ in crude fat. Therefore, *r* of *D*_60_ was not further examined. The *r* of crude fiber and crude fat is negatively correlated with *D*_T_ and is not consistent. The *r* of moisture and total carbohydrate are highly correlated only with *D*_70_. The *r* of ash and crude protein in swill with *D*_70_, *D*_75_, and *D*_80_ are moderate and more consistent. Therefore, in this study, ASFV in swill formula with higher ash and crude protein is more resistant to temperature.

## Discussion

The OIE suggested the thermal inactivation of ASFV in swill by maintaining a temperature higher than 90°C for more than 60 min (11), whereas the FAO suggested the heat treatment of swill to inactivate ASFV at 70°C for 30 min (3). Scientific evidence is then required to resolve such inconsistent practices for smallholder pig farmers. Therefore, this study is dedicated to systematically investigate the thermal inactivation of ASFV in three swill formulae with different nutritive compositions and, also, to develop a *D*_T_ model to predict *D*_T_ for some other inactivation temperatures.

The virucidal activity of heat treatment of ASFV in swill is illustrated by the significant reduction of ASFV titer at all inactivation temperatures tested as shown in Figure 1. The most pronounced effect of the heat treatment against ASFV in swill is at the highest inactivation temperature of 80°C in swill since the ASFV titers drop fastest within the shortest period of inactivation time.

In this study, *D*_T_ was used as a comparative index of heat resistance of ASFV across inactivation temperatures in swill for an equivalent 1–log reduction. Note that *D*_T_ at a higher temperature is theoretically less than *D*_T_ at a lower temperature (16, 17) since a higher inactivation temperature takes a shorter inactivation time to achieve the same 1–log reduction. As expected, the mean *D*_80_ of three swill formulae is significantly the lowest, while the mean *D*_60_ of three swill formulae is significantly highest in Table 3. Therefore, this indicates that virucidal activity of ASFV in swill is temperature dependent (temperature effect). This result is also supported by the fact that the DRT curves of ASFV across 4 inactivation temperatures are significantly different from zero. Therefore, in general, smallholder pig farms could shorten the heat treatment time of swill easily by increasing the temperature of heat treatment.

A previous study correlated moisture content with *D*_25_ of transmissible gastroenteritis virus (TGEV), porcine epidemic diarrhea virus (PEDV), and porcine delta coronavirus (PDCoV) in complete feed and several feed ingredients, such as spray-dried porcine plasma, meat meal, meat, bone meal, blood meal, soybean meal, and corn (18). The result indicated that *r* of moisture was positively correlated with *D*_25_ of TGEV and PDCoV, which are 0.41 (*p* = 0.03) and 0.48 (*p* = 0.01), respectively. This indicates that TGEV and PDCoV in moist feed ingredients are more heat resistant than those in dry feed ingredients. The moisture effect in the previous study was only compatible with *r* of *D*_70_ of ASFV in swill at 0.72 in this study (Table 5). However, in this study, the correlation of ash and protein in swill with *D*_70_, *D*_75_, and *D*_80_ was moderate and more consistent (Table 5). This indicates that ASFV in swill formula with higher ash and crude protein is more heat resistant. The effect of different swill compositions on the heat resistance of ASFV in the swill of the previous study and this study might be explained by the different inactivation temperatures (lower vs. higher than 60°C), study matrices, and viruses (19, 20). After all, the statistical analysis of heat resistance of ASFV in swill across 4 inactivation temperatures demonstrated that *D*_T_ of ASFV in three swill formulae for each inactivation temperature was not significantly different. In other words, statistically, the heat resistance of ASFV in swill was not affected by the nutritive composition of swill formulae at all. Therefore, the *D*_60_, *D*_70_, *D*_75_, and *D*_80_ in this study could be widely used for any nutritive composition of swill other than the three swill formulae in this study.

The significant reduction of ASFV in swill occurred in the range 60–80°C (Table 2). Therefore, according to this study, the effective heat treatment for ASFV in swill is at least 60°C. Now that this study demonstrated the starting inactivation temperature at 60°C, the next parameter of the heat treatment is the inactivation time. This parameter is a product of two factors, which are *D*_60_ and the required log reduction of ASFV in swill. The highest mean *D*_60_ at 33.47 min/log in swill formula 2 was chosen (Table 3). The required log reduction of ASFV in swill is determined by the ASFV titer in the swill. This information is not available and some pork or pork products were part of the table scrap or food waste, and so the ASFV load in some pork products was chosen instead. The highest titers of ASFV in a lymph node, bone marrow, blood, pork, and fat have been previously reported as 10.9, 10.9, 9.9, 7.7, and 7.4 log plaque-forming units (PFU)/g (21). In the worst-case scenario, where the highest heat resistance of ASFV in three swill formulae and the highest titer of pork products reported was assumed, the inactivation time of ASFV at 60°C was the product of 33.47 min/log and 10.9 log, which is approximately 365 min or 6 h. Likewise, the calculated inactivation times to eliminate ASFV titer contaminated in swill at 70, 75, and 80°C based on *D*_T_ of ASFV in swill Formula 2 are shown in Table 6.

**Table 6:** Calculated inactivation time to eliminate ASFV contaminated in the swill Formula 2.

**Inactivation**	**Mean**	**Highest ASF titer**	**Inactivation**
**temp. (**°**C)**	***D*** **_T_ (min)**	**(log PFU/g)**	**time (min)**
60	23.22	10.9	253
70	10.91	10.9	119
75	2.22	10.9	24
80	1.47	10.9	16
90	0.35	10.9	4
100	0.08	10.9	1

It is noteworthy to examine the inactivation time recommended by the FAO to eliminate ASFV contaminated in swill at 70°C in comparison with the inactivation time calculated by *D*_70_ of ASFV in swill in this study, coupled with the highest titer of pork products reported (21) as shown in Table 6. The calculated inactivation time for ASFV contaminated in swill at 70°C in this study was 119 min, which is about 4 times more than the inactivation time suggested by the FAO.

Since the inactivation temperatures of ASFV in swill formulae in this study are in the range 60–80°C, the *D*_90_ and *D*_100_ of ASFV in swill were not available but could be predicted by the *D*_T_ model in this study (Table 4). Since the mean *D*_70_, *D*_75_, and *D*_80_ of ASFV in swill Formula 2 were highest, the *D*_90_ and *D*_100_ based on the *D*_T_ model of ASFV in swill Formula 2 were 0.35 and 0.08 min, respectively. Similar to the calculated inactivation time at 70°C in the previous paragraph, the calculated inactivation times for ASFV contaminated in swill at 90 and 100°C in this study were ~4 and 1 min, respectively (Table 6), while the inactivation temperature and time to eliminate ASFV contaminated in swill suggested by the OIE were at least 90°C for at least 60 min. Even though this study proposed an approach to determine the inactivation time based on the *D*_T_ and highest ASFV titer in the swill, the inconsistencies of the inactivation time of the FAO, the OIE, and this study might be due to some factors such as the virus strains, feed ingredient matrices, inactivation models, and the assumption of highest ASFV titer in the swill (3, 11, 16–18).

Some smallholder pig farmers could not afford the thermometer measuring swill temperature while the boiling temperature of swill could be visually determined without using any device. Therefore, the practical approach for smallholder pig farmers is setting the boiling temperature of the swill as the target cooking temperature to inactivate ASFV in the swill. Since the *D*_T_ models against ASFV in swill demonstrated in this study are complicated and prone to error, this study provides a spreadsheet as a fast and convenient tool to predict the cooking times of swill with 95% confidence intervals based on *D*_T_ models of 3 swill formulae in Table 4 by just entering the target cooking temperature and desire log reduction of ASFV. Note that the *D*_T_ models were fitted with high levels of *r*^2^, and then these models were valid in the temperature range of this study. The cooking times of swill predicted by *D*_T_ models outside the study temperatures should be used with caution. The spreadsheet so-called “EZ Swill Cooking Time,” inactivating ASFV in swill, is free to download from the link in the Supplementary Materials.

## Conclusion

The thermal inactivation of ASFV in three swill formulae was investigated. The effective inactivation temperatures of ASFV in swill were at least 60°C. The rate of thermal inactivation was represented by the *D*_T_ or time required to reduce ASFV per 1 log at an inactivation temperature (*T*). The mean *D*_60_, *D*_70_, *D*_75_, and *D*_80_ of all swill formulae are in the ranges 23.21–33.47, 5.83–10.91, 2.15–2.22, and 1.36–1.47 min, respectively. This *D*_T_ could be widely used for any nutritive composition of swill other than three swill formulae in this study since there was no statistical difference between the *D*_T_ of ASFV across 3 swill formulae. Based on *D*_70_ and the predicted *D*_90_ from the *D*_T_ model in this study, including the highest ASFV titer in pork products, the calculated inactivation times at 70 and 90°C were 119 and 4 min, respectively.

## Data Availability Statement

The datasets presented in this study can be found in online repositories. The names of the repository/repositories and accession number(s) can be found in the article/Supplementary Material.

## Author Contributions

SN conceptualized and designed the overall study. TS, PB, NS, and WL performed the experiment collected and analyzed the data. SN and TS drafted the manuscript. PB and CN edited the manuscript. All authors contributed to the article and approved the submitted version.

## Funding

This study was supported by Agricultural Research Development Agency (Public Organization).

## Conflict of Interest

The authors declare that the research was conducted in the absence of any commercial or financial relationships that could be construed as a potential conflict of interest.

## Publisher's Note

All claims expressed in this article are solely those of the authors and do not necessarily represent those of their affiliated organizations, or those of the publisher, the editors and the reviewers. Any product that may be evaluated in this article, or claim that may be made by its manufacturer, is not guaranteed or endorsed by the publisher.
