# Supplementary material for: Thermal Inactivation of African Swine Fever Virus in Swill
Source: Front Vet Sci. 2022 Jun 6;9:906064. doi: 10.3389/fvets.2022.906064 (PMC9207410; doi:10.3389/fvets.2022.906064)
Supplement: Supplementary file 1 [file Data_Sheet_1.PDF]

# Predicting *D* value and cooking time for swill

Version 1.01 :  Copyright 2022 Suphachai Nuanualsuwan

This spreadsheet was initially generated as the data availability to the publication article;
Thermal inactivation of African swine fever virus in swill

**Frontiers in Veterinary Science**

**Version 1.01**

Suphachai Nuanualsuwan
Center of Excellence for Food and Water Risk Analysis (FAWRA)
Department of Veterinary Public Health, Faculty of Veterinary Science,
Chulalongkorn University, Bangkok, Thailand.
email: suphachai.n@chula.ac.th

**Acknowledgment**

This spreadsheet was inspired by the comment from reviewers of Frontiers in Veterinary Science

**Disclaimer**

The author has taken every care to ensure that the output from this spreadsheet is accurate.

The objetive of this spreadsheet is to predict the *D* values and then the cooking time of 3 swill formulae.

Neither the author nor Journal accept any liability for any consequences, direct or indirect resulting from a decision by the user to take, or not to take, any action based on outputs from this spreadsheet.

This spreadsheet was designated to predict the mean *D* value and cooking time of 3 swill formulae.

| **Swill composition** | **Percent (w/w)[a]** | | |
|---|---|---|---|
| | **Swill formula 1** | **Swill formula 2** | **Swill formula 3** |
| Crude fiber | 5.42 ± 1.23 | 0.59 ± 0.21 | 0.50 ± 0.11 |
| Crude Fat | 0.98 ± 0.14 | 2.99 ± 0.42 | 4.43 ± 0.69 |
| Moisture | 0.98 ± 0.14 | 72.82 ± 2.42 | 70.44 ± 0.21 |
| Total Carbohydrate | 15.21 ± 2.49 | 25.38 ± 2.21 | 21.71 ± 0.12 |
| Ash | 0.57 ± 0.04 | 1.82 ± 0.42 | 0.76 ± 0.05 |
| Crude Protein | 3.22 ± 0.37 | 7.96 ± 0.25 | 2.74 ± 0.13 |

# Thermal inactivation of African swine fever virus in swill

**Swill formula 1: 5.42% Crude fiber, 0.98% Crude Fat, 15.21% Total Carbohydrate, 3.22% Crude Protein**

| Celcius ($^{o}$C) | Farenheit ($^{o}$F) | **Predicted *D* value** | | | Desired Log reduction | **Predicted Cooking time (min)** | | | Add Safety margin |
|---|---|---|---|---|---|---|---|---|---|
| | | Lower 95% CI | Mean | Upper 95% CI | | Lower 95% CI | Mean | Upper 95% CI | |
| 60 | 140 | 12 | 28 | 64 | 4 | 48 | 110 | 255 | 383 |
| 70 | 158 | 2 | 6 | 15 | 4 | 9 | 23 | 61 | 92 |
| 80 | 176 | 0.4 | 1.2 | 3.7 | 4 | 1.6 | 4.8 | 14.7 | 22 |
| 90 | 194 | 0.1 | 0.3 | 0.9 | 4 | 0.3 | 1.0 | 3.5 | 5 |
| 100 | 212 | 0.01 | 0.1 | 0.2 | 4 | 0.05 | 0.2 | 0.9 | 1.3 |

Version 1.01 : Copyright 2022 Suphachai Nuanualsuwan
CI: Confidence interval
Adding safety of margin by 50%

**Instructions**

1. Enter cooking temperature (celcius) of swill in cell B14
2. Enter desired log reduction in cell G14

# Thermal inactivation of African swine fever virus in swill

**Swill formula 2: 0.59% Crude fiber, 2.99% Crude Fat, 25.38% Total Carbohydrate, 7.96% Crude Protein**

| Celcius (°C) | Farenheit (°F) | Predicted *D* value | | | Desired Log reduction | Predicted Cooking time (min) | | | Add Safety margin |
|---|---|---|---|---|---|---|---|---|---|
| | | Lower 95% CI | Mean | Upper 95% CI | | Lower 95% CI | Mean | Upper 95% CI | |
| 60 | 140 | 3 | 28 | 258 | 5 | 16 | 142 | 1291 | 1936 |
| 70 | 158 | 0.5 | 7 | 86 | 5 | 2 | 33 | 429 | 644 |
| 80 | 176 | 0.1 | 2 | 29 | 5 | 0.4 | 7.5 | 143 | 214 |
| 90 | 194 | 0.01 | 0.3 | 9.5 | 5 | 0.1 | 1.7 | 48 | 71 |
| 100 | 212 | 0.002 | 0.1 | 3 | 5 | 0.01 | 0.4 | 16 | 23.7 |

Version 1.01 : Copyright 2022 Suphachai Nuanualsuwan

CI: Confidence interval

Adding safety of margin by 50%

**Instructions**

1. Enter cooking temperature (celcius) of swill in cell B14
2. Enter desired log reduction in cell G14

# Thermal inactivation of African swine fever virus in swill

**Swill formula 3: 0.5% Crude fiber, 4.43% Crude Fat, 21.71% Total Carbohydrate, 2.74% Crude Protein**

| Celcius ($^oC$) | Farenheit ($^oF$) | Predicted *D* value | | | Desired Log reduction | Predicted Cooking time (min) | | | Add Safety margin |
|---|---|---|---|---|---|---|---|---|---|
| | | Lower 95% CI | Mean | Upper 95% CI | | Lower 95% CI | Mean | Upper 95% CI | |
| 60 | 140 | 7 | 38 | 201 | 4 | 29 | 151 | 805 | 1207 |
| 70 | 158 | 1 | 7 | 49 | 4 | 4 | 28 | 196 | 294 |
| 80 | 176 | 0.1 | 1.3 | 12 | 4 | 0.6 | 5.2 | 48 | 72 |
| 90 | 194 | 0.02 | 0.2 | 2.9 | 4 | 0.1 | 1.0 | 12 | 18 |
| 100 | 212 | 0.003 | 0.04 | 0.7 | 4 | 0.01 | 0.2 | 2.8 | 4.3 |

**Instructions**

1. Enter cooking temperature (celcius) of swill in cell B14
2. Enter desired log reduction in cell G14

Version 1.01 : Copyright 2022 Suphachai Nuanualsuwan

CI: Confidence interval

Adding safety of margin by 50%

| | |
|---|---|
| Password to unlock protection sheet | 1234 |
